# Somatostatin Receptor Splicing Variant *sst5TMD4* Overexpression in Glioblastoma Is Associated with Poor Survival, Increased Aggressiveness Features, and Somatostatin Analogs Resistance

**DOI:** 10.3390/ijms23031143

**Published:** 2022-01-20

**Authors:** Antonio C. Fuentes-Fayos, Miguel E. G-García, Jesús M. Pérez-Gómez, Annabel Peel, Cristóbal Blanco-Acevedo, Juan Solivera, Alejandro Ibáñez-Costa, Manuel D. Gahete, Justo P. Castaño, Raúl M. Luque

**Affiliations:** 1 Maimonides Biomedical Research Institute of Cordoba (IMIBIC), 14004 Cordoba, Spain; b22fufaa@uco.es (A.C.F.-F.); b62gagam@uco.es (M.E.G.-G.); b42pegoj@uco.es (J.M.P.-G.); crichess@hotmail.com (C.B.-A.); juan.solivera@gmail.com (J.S.); b12ibcoa@uco.es (A.I.-C.); bc2gaorm@uco.es (M.D.G.); justo@uco.es (J.P.C.); 2 Department of Cell Biology, Physiology, and Immunology, University of Cordoba, 14004 Cordoba, Spain; 3 Reina Sofia University Hospital (HURS), 14004 Cordoba, Spain; 4 CIBER Physiopathology of Obesity and Nutrition (CIBERobn), 14004 Cordoba, Spain; 5 School of Biosciences, Cardiff University, Sir Martin Evans Building, Museum Ave, Cardiff CF10 3AX, UK; annabel.peel@gmail.com; 6 Department of Neurosurgery, Reina Sofia University Hospital, 14004 Cordoba, Spain

**Keywords:** sst5TMD4, somatostatin receptor, splicing variant, glioblastoma, somatostatin analogs

## Abstract

Glioblastoma (GBM) is the most malignant and lethal brain tumor. Current standard treatment consists of surgery followed by radiotherapy/chemotherapy; however, this is only a palliative approach with a mean post-operative survival of scarcely ~12–15 months. Thus, the identification of novel therapeutic targets to treat this devastating pathology is urgently needed. In this context, the truncated splicing variant of the somatostatin receptor subtype 5 (sst5TMD4), which is produced by aberrant alternative splicing, has been demonstrated to be overexpressed and associated with increased aggressiveness features in several tumors. However, the presence, functional role, and associated molecular mechanisms of sst5TMD4 in GBM have not been yet explored. Therefore, we performed a comprehensive analysis to characterize the expression and pathophysiological role of sst5TMD4 in human GBM. sst5TMD4 was significantly overexpressed (at mRNA and protein levels) in human GBM tissue compared to non-tumor (control) brain tissue. Remarkably, *sst5TMD4* expression was significantly associated with poor overall survival and recurrent tumors in GBM patients. Moreover, in vitro *sst5TMD4* overexpression (by specific plasmid) increased, whereas *sst5TMD4* silencing (by specific siRNA) decreased, key malignant features (i.e., proliferation and migration capacity) of GBM cells (U-87 MG/U-118 MG models). Furthermore, *sst5TMD4* overexpression in GBM cells altered the activity of multiple key signaling pathways associated with tumor aggressiveness/progression (AKT/JAK-STAT/NF-κB/TGF-β), and its silencing sensitized GBM cells to the antitumor effect of pasireotide (a somatostatin analog). Altogether, these results demonstrate that sst5TMD4 is overexpressed and associated with enhanced malignancy features in human GBMs and reveal its potential utility as a novel diagnostic/prognostic biomarker and putative therapeutic target in GBMs.

## 1. Introduction

Glioblastoma (GBM) is the most prevalent and malignant brain tumor in adults, with the highest mortality among the Central Nervous System (CNS) tumors [1,2]. Current standard treatment consists of surgery as first line approach followed by or combined with radiotherapy and/or chemotherapy depending on various clinical factors (patient’s age and sex, type and degree of tumor, location and size, etc.) [3,4,5]. However, this is only a palliative approach with a mean post-operative survival of ~12–15 months from diagnosis, and a 5-year survival rate lower than 6% [3,4,6]. Therefore, a more profound knowledge of GBM biology is urgently required to discover and identify novel and effective diagnosis/prognosis biomarkers and therapeutic targets.

We have recently demonstrated that the dysregulation of the alternative splicing process could represent a valuable source for the identification of novel diagnostic, prognostic, and therapeutic targets in different tumor pathologies including GBM [7,8,9,10]. In this context, somatostatin receptors belong to a complex family of different G-protein coupled receptors with seven transmembrane domains (SSTR1-5), which show a similar structure and share common signaling mechanisms [11]. Interestingly, we have demonstrated that the complexity of the somatostatin system in humans is increased by the existence of two variants of *SSTR5* with four and five transmembrane domains (sst5TMD4 and sst5TMD5, respectively) generated by alternative splicing [12,13]. Specifically, sst5TMD4 has been shown to be overexpressed in several hormone-related tumors compared with non-tumor tissues, where it enhanced aggressiveness features [14,15,16,17,18,19,20,21,22]. Moreover, sst5TMD4 has been shown to reduce the response to somatostatin analogs (SSAs; e.g., octreotide) [14,17,18,22,23], which are used as valuable drugs to treat different tumor pathologies, including pituitary and neuroendocrine tumors [24,25]. Unfortunately, attempts to use SSAs as medical therapy in brain tumors have rendered controversial results since some of the available studies have not reported a clear therapeutic value for SSAs; however, the mechanistic reasons underlying those experimental failures remain unknown [26,27,28,29,30].

For all the reasons mentioned above, this study was aimed at investigating, for the first time, the presence, functional role, and mechanisms of actions of the *sst5TMD4* splice variant and its truncated protein in GBM. To that end, we applied different experimental approaches in GBM tissues/cells including the analysis of sst5TMD4 in human samples (GBM and control brain tissues) and its potential association with relevant clinical data (survival, recurrence, mutations, etc.), as well as the consequences of the modulation of *sst5TMD4* levels (overexpression and silencing) in different key functional parameters of aggressiveness (proliferation and migration), signaling pathways associated with tumor aggressiveness/progression, and in the response to SSAs (octreotide, lanreotide, and pasireotide).

## 2. Results

### 2.1. sst5TMD4 Levels Are Significantly Overexpressed in GBM Samples and Associated with Poor Prognosis and Survival Rate in Patients with GBM

**Figure 1:**
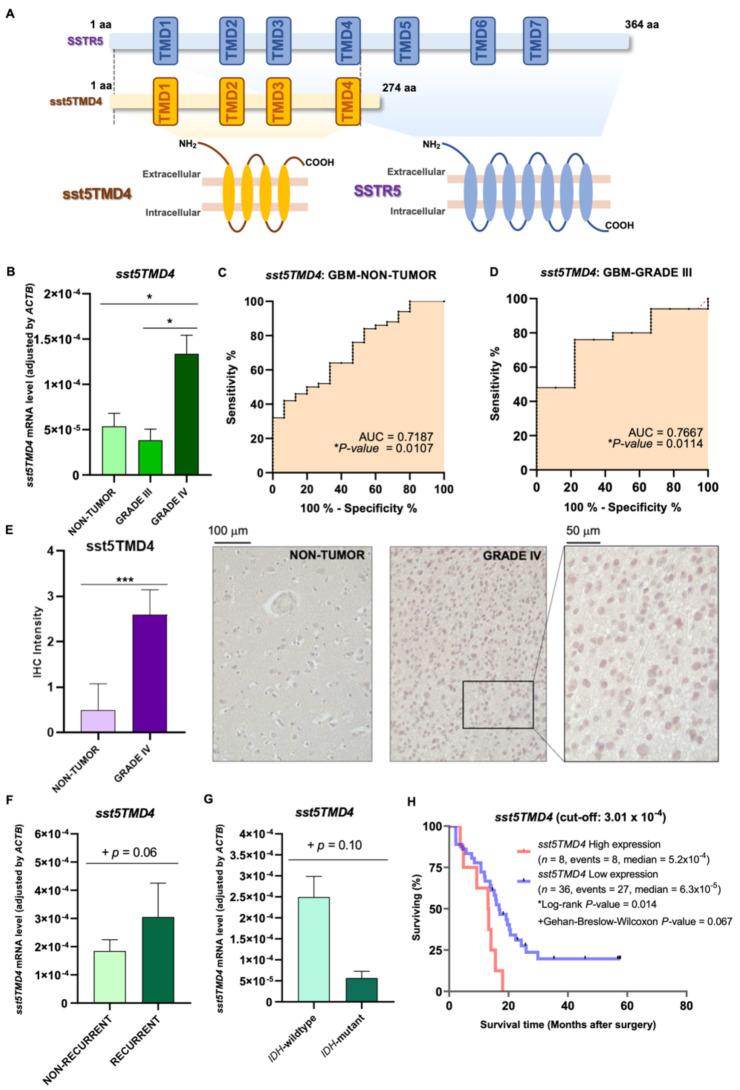
Characterization and clinical relevance of *sst5TMD4* in glioblastoma samples. (**A**) Schematic comparison of transmembrane domains (TMDs) and structure between SSTR5 and sst5TMD4 (dot lines delimit the homologous region between both aminoacidic sequences). (**B**) *sst5TMD4* mRNA expression level in an internal cohort of patients [GBM (*n* = 47) grade III anaplastic astrocytoma (*n* = 9); non-tumor/control brain tissues (*n* = 20)]. (**C**) ROC-curve analysis comparing mRNA expression of *sst5TMD4* in GBM vs. non-tumor tissues (associated AUC is also indicated). (**D**) ROC-curve analysis comparing mRNA expression of *sst5TMD4* in GBM vs. grade III anaplastic astrocytomas (associated AUC is also indicated). (**E**) sst5TMD4-immunohistochemistry staining in non-tumor (*n* = 4) and grade IV/GBM (*n* = 5) samples. Representative images are included. (**F**) Comparative of *sst5TMD4* mRNA expression levels between *IDH*-wildtype (*n* = 38) and *IDH*-mutant (*n* = 5) GBMs, and (**G**) between recurrent *(n* = 41) and non-recurrent (*n* = 9) GBMs. (**H**) Kaplan–Meier survival curve discerning between GBM patients with high and low expression levels of *sst5TMD4* from our cohort of patients. Data represent means ± SEM. (*) *p* < 0.05, (***) *p* < 0.001 significantly differs from control samples; (+) existence of a statistic tendency (*p* ≤ 0.10).

Key demographic and clinical characteristics of the patients included in this study are summarized in Table 1. Expression levels of *sst5TMD4* (Figure 1A) were found to be significantly higher in samples from GBM patients (*n* = 47; grade IV astrocytoma) compared to anaplastic astrocytoma samples (*n* = 9; grade III anaplastic astrocytoma; a brain tumor subtype that is notably less aggressive than GBM [31]) (Figure 1B), and to non-tumor brain samples [*n* = 20; 16 samples from autopsies (4 donor individuals/4 different brain areas: Brocca, Wernicke, cingulate, and medial) and 4 samples from surgery of epileptic patients, which showed similar levels of expression of *sst5TMD4* (Supplemental Figure S1A)] (Figure 1B). Moreover, this differential expression was corroborated by Receiver Operative Characteristic (ROC) analyses since *sst5TMD4* mRNA levels was able to significantly discriminate between GBM vs. non-tumor samples (Figure 1C), and between GBM vs. grade III astrocytoma samples (Figure 1D), with an Area Under the Curve (AUC) of 0.7187 and 0.7667, respectively. Furthermore, an immunohistochemistry (IHC) analysis in a representative cohort of available Formalin-Fixed Paraffin-Embedded (FFPE) samples [GBM (*n* = 5) and non-tumor (*n* = 4) samples] using a specific antibody previously designed and validated by our group [12,22] confirmed the overexpression at protein level of *sst5TMD4* in GBM tissues (cytoplasmic and nuclear staining) compared with non-tumor tissues (Figure 1E).

To further investigate the pathophysiological implication of sst5TMD4 in GBM, we next studied its potential association with relevant clinical parameters. We found that the presence of the mutation in isocitrate dehydrogenase 1 (*IDH1*mut, which is classically linked with better prognosis, survival, and low-level necrosis in gliomas [32]), tends to be associated with low *sst5TMD4* expression (*p* = 0.10; Figure 1F). Similarly, recurrent tumors tend to express higher levels of *sst5TMD4* compared to non-recurrent tumors (*p* = 0.06; Figure 1G). Most notably, we demonstrated a significant clinical association between a better survival rate in GBM patients with low expression levels of *sst5TMD4* compared with patients with higher *sst5TMD4* expression (Figure 1H). No correlation was found between *sst5TMD4* mRNA levels and other available clinical data (*TP53* mutations, previous pathologies, brain impairments, etc.; Supplemental Figure S1B).

### 2.2. Overexpression of sst5TMD4 Increases Aggressiveness Parameters in GBM Cells

**Figure 2:**
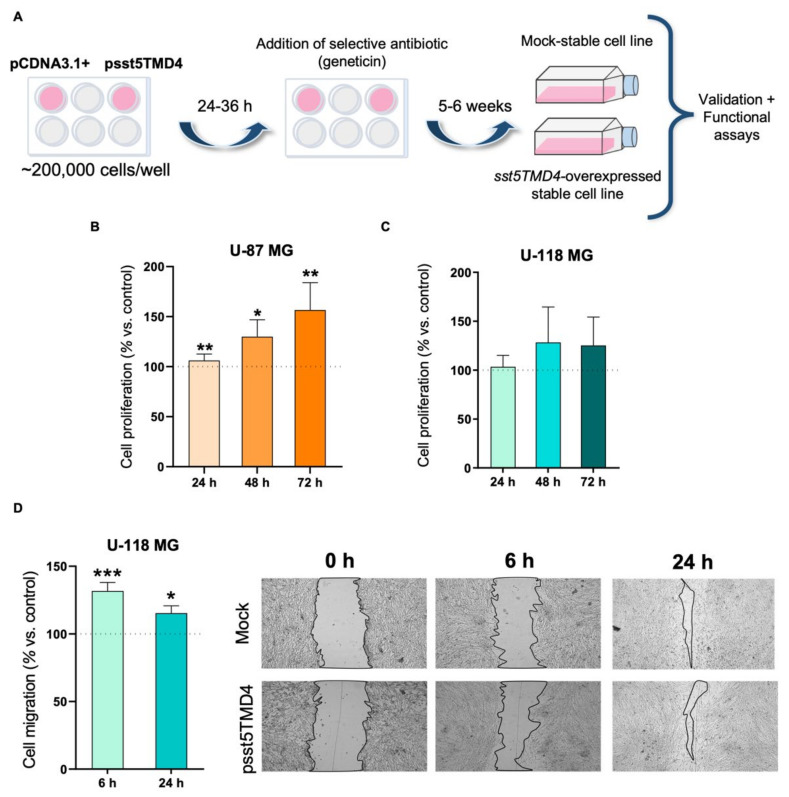
Stable *sst5TMD4* overexpression increases oncogenic parameters in vitro in glioblastoma cells. (**A**) Schematic workflow to obtain stable overexpressed *sst5TMD4* U-87 MG and U-118 MG cell lines. Proliferation rate of *sst5TMD4*-overexpressed cells compared to control (mock-transfected cells; pointed line) in U-87 MG (**B**) and in U-118 MG (**C**) (*n* = 3). (**D**) Migration rate of *sst5TMD4*-overexpressed U-118 MG cells compared to control (mock-transfected cells; pointed line; *n* = 3). Representative images of the migration capacity are also included. Data represent means ± SEM. (*) *p* < 0.05, (**) *p* < 0.01, (***) *p* < 0.001 significantly differ from control samples.

To examine the functional role of sst5TMD4 in the proliferation and migration of GBM cells, we generated stably-transfected cells from two metabolically different GBM cell lines: U-87 MG and U-118 MG (Figure 2A) [33]. These studies indicated that *sst5TMD4* stable overexpression (Supplemental Figure S2A) significantly increased the proliferation rate of U-87 MG cells at 24, 48 and 72 h (Figure 2B) compared to cells transfected with a mock plasmid (used as control), while it numerically (albeit not significantly) increased proliferation of U-118 MG cells, especially at 48 and 72 h (Figure 2C). Cell migration was also evaluated in U-118 MG but not in U-87 MG cells [due to the inability of U-87 MG cells to migrate in these conditions (i.e., growth in clusters when the confluence is >75% [8])] and found that *sst5TMD4* overexpression also significantly increased migration rate of U-118 MG cells, at 6 and 24 h after the wound was performed (Figure 2D).

### 2.3. Overexpression of sst5TMD4 Modulated the Phosphorylation Levels of Different Proteins Associated with Key Oncogenic Pathways in GBM Cells

**Figure 3:**
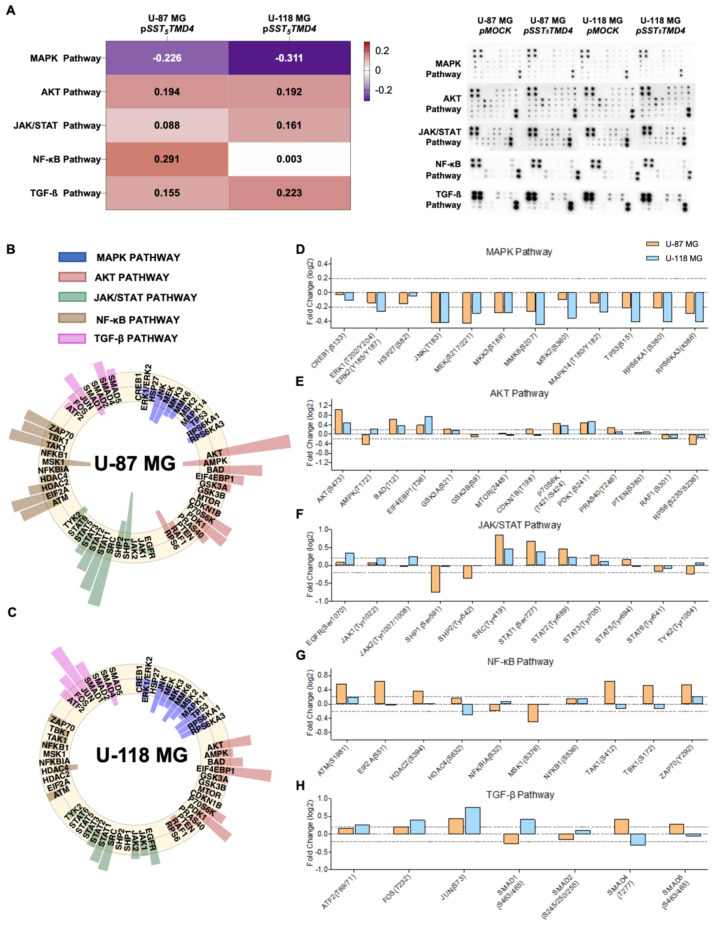
*sst5TMD4* overexpression induces phosphorylation of key oncogenic pathway components in a cell line dependent manner. (**A**) Heatmap showing the logarithm of fold-change mean corresponding to each signaling pathway comparing *sst5TMD4*-transfected (overexpression) vs. mock-transfected cells (Left panel); Membranes showing the spots quantified in order to study the phosphorylation level of 55 proteins under different experimental conditions (*sst5TMD4*-transfected vs. mock-transfected cells; Right panel). (**B**,**C**) Circle plot with Log2 (Fold Change; FC) of each measured protein in the phosphoarray comparing *sst5TMD4*-transfected (overexpression) vs. mock-transfected cells [U-87 MG (**B**) and U-118 MG (**C**)]. (**D**–**H**) Individual phosphorylation protein level after *sst5TMD4* overexpression vs. the control condition in both GBM cell lines [threshold: log2 (FC) = ± 0.2; pointed line].

Phosphoprotein analysis in both GBM cell models (U-87 MG and U-118 MG) uncovered different molecular pathways that could be involved in the observed oncogenic actions of sst5TMD4 in GBM cells. Specifically, overexpression of *sst5TMD4* in U-87 MG and U-118 MG cells induced an overall inactivation of the MAPK pathway and an overall activation of the AKT, JAK/STAT and TGF-β pathways compared with control (mock-transfected) cells (Figure 3A), although some precise changes in the phosphorylation pattern of proteins belonging to these and other signaling pathways were found to differ depending on the particular cell model analyzed (i.e., activation of NF-κB in U-87 MG but not in U-118 MG cells; Figure 3A–C).

In more detail, *sst5TMD4* overexpression produced a clear reduction in the phosphorylation levels of multiple proteins of the MAPK pathway, which seemed to be more pronounced in U-118 MG compared with U-87 MG cells (Figure 3A). Specifically, an inhibition of JNK, MEK, MMK3, MMK6, TP53, RPS6KA1, and RPS6KA3 levels was observed in both cell models, while ERK1/2, MSK2, and MAPK14 levels were reduced only in U-118 MG cells (statistically significant changes [Log2 (Fold Change) is ≥0.2/≤0.2]; Figure 3B–D). In addition, *sst5TMD4* overexpression induced an increase in the phosphorylation levels of several proteins belonging to the AKT pathway in both GBM cell models (Figure 3A), including AKT1, BAD, EIF4EBP1, P70S6K, and PDK1, as well as an increase in GSK3A, CDKN1B, and PRASA40 only in U-87 MG (Figure 3B,C,E). Interestingly, a significant reduction in the phosphorylation levels of RPS6 in U-87 MG was also observed, while the levels of AMPK were oppositely regulated in these two GBM cell models (i.e., decreased in U-87 MG and increased in U-118 MG cells) (Figure 3B,C,E). Regarding JAK/STAT pathway, a significant increase in the phosphorylation levels of upstream proteins (i.e., EGFR, JAK1, and JAK2) was specifically found only in U-118 MG (Figure 3C,F). Furthermore, we also observed that the phosphorylation ratio of SRC, STAT1, STAT2 and STAT3 was increased by ~two-fold in U-118 MG vs. U-87 MG in response to *sst5TMD4* overexpression (Figure 3B,C,F). Moreover, a reduction in the phosphorylation levels of SHP-1, SHP-2 and TYK2 was found only in U-87 MG (Figure 3B,F). Notably, *sst5TMD4* overexpression significantly altered the phosphorylation levels of several proteins of the NF-κB pathway in U-87 MG but not in U-118 MG cells (Figure 3A) (i.e., robust activation of ATM, EIF2A, HDAC2, TAK1, TBK1, and ZAP70, and inactivation of MSK1; Figure 3B,C,G). In fact, only a slight activation of ZAP70 and inactivation of HDAC4 was found in U-118 MG cells (Figure 3C,G). Finally, *sst5TMD4* overexpression resulted in a higher activation of the TGF-β pathway in U-118 MG compared to U-87 MG cells (Figure 3A). Specifically, a significant hyperphosphorylation in ATF2, FOS, JUN, SMAD1 levels and an inactivation in SMAD4 were observed in U-118 MG cells (Figure 3C,H), while a less significant activation in FOS and JUN levels together with a specific increase in SMAD4 and SMAD5 levels was observed in U-87 MG cells (Figure 3B,H).

### 2.4. Silencing of sst5TMD4 Reduces Aggressiveness Parameters in GBM Cells

**Figure 4:**
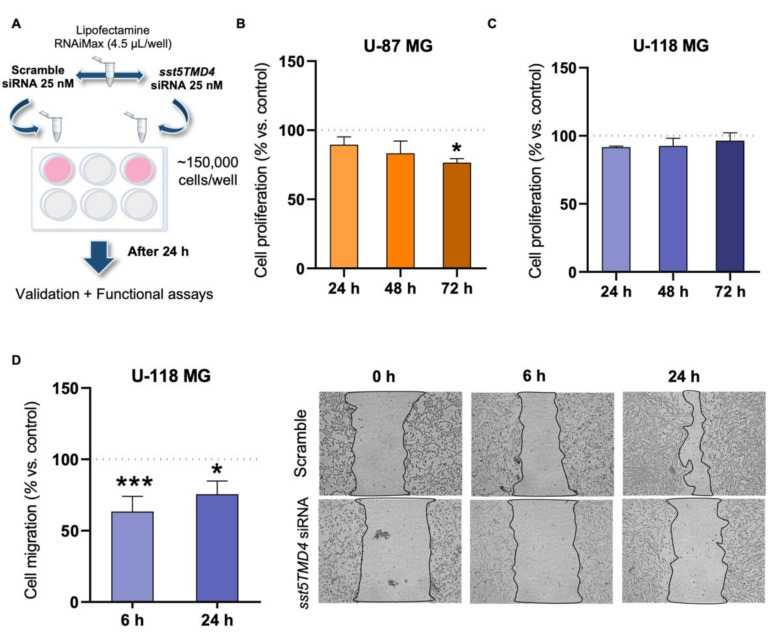
*sst5TMD4* silencing promotes the reduction of oncogenic features in vitro in glioblastoma cells. (**A**) Schematic diagram followed to obtain silenced *sst5TMD4* GBM cells (U-87 MG and U-118 MG). (**B**,**C**) Proliferation rate of *sst5TMD4*-silenced cells compared to control (scramble-transfected cells; pointed line) in U-87 MG (**B**) and U-118 MG (**C**) (*n* = 3). (**D**) Migration rate of *sst5TMD4*-overexpressed U-118 MG cells compared to control (mock-transfected cells; pointed line; *n* = 3). Representative images of the migration capacity are also included. Data represent means ± SEM. (*) *p* < 0.05, (***) *p* < 0.001 significantly differ from control samples.

In order to examine whether a reduction in the expression of *sst5TMD4* in GBM cells could be of interest from a therapeutic point of view, we next determined the proliferation and migration rate of GBM cells in response to the specific silencing of *sst5TMD4* levels by a validated siRNA (Figure 4A and Supplemental Figure S2B). These results indicated that *sst5TMD4* silencing significantly decreased the proliferation rate of U-87 MG, but not U-118 MG, comparing cells transfected with a scramble control siRNA (Figure 4B,C). Moreover, *sst5TMD4* silencing significantly decreased cell migration capacity of U-118 MG at 6 and 24 h after the wound was performed (Figure 4D).

### 2.5. Overexpression of sst5TMD4 Alters the Basal Expression of SSTR2 in GBM Cells and Sensitized the Response of GBM Cells to Pasireotide Treatment

**Figure 5:**
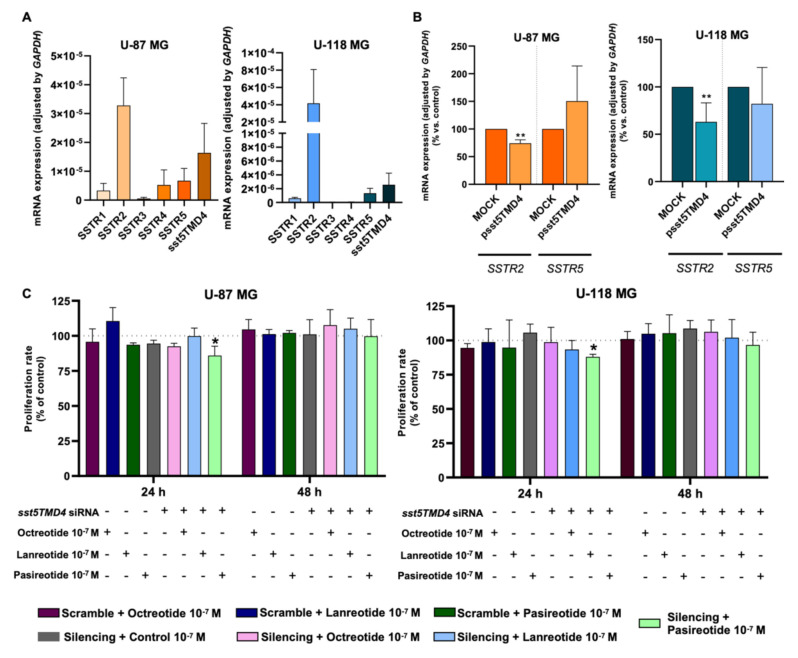
Overexpression of *sst5TMD4* alters the basal expression of *SSTR2* in GBM cells and sensitized the response of GBM cells to pasireotide treatment. (**A**) Basal expression profile (mRNA levels) of somatostatin receptor subtypes (*SSTR1-5*) in U-87 MG and U-118 MG cell lines (*n* = 3). (**B**) mRNA levels of *SSTR2* and *SSTR5* after *sst5TMD4* overexpression (vs. control; pointed line) in U-87 MG and U-118 MG cell lines (*n* = 4). (**C**) Comparison of the proliferation rate of U-87 MG and U-118 MG in response to the treatment with different somatostatin analogs (octreotide, lanreotide or pasireotide) under basal-normal conditions (scramble control cells; pointed line) vs. *sst5TMD4*-silenced cells (*n* = 3). Data represent means ± SEM. (*) *p* < 0.05, (**) *p* < 0.01 significantly differ from control samples.

A differential expression level was found for each of the SSTR subtypes in the GBM cell models (Figure 5A). Specifically, the present work revealed that *SSTR2* is the predominant SSTR subtype expressed in GBM cells [absolute mRNA copy number (normalized mean ± SEM): 3.2 × 10^−5^ ± 9.6 × 10^−6^ and 4.2 × 10^−5^ ± 3.9 × 10^−5^ in U-87 MG and U-118 MG, respectively], followed by *sst5TMD4* ≳ *SSTR5* ≳ *SSTR4* ≳ *SSTR1 > SSTR3* in U-87 MG (1.6 × 10^−5^ ± 8.3 × 10^−6^; 6.7 × 10^−6^ ± 4.2 × 10^−6^; 5.3 × 10^−6^ ± 5.2 × 10^−6^; 3.3 × 10^−6^ ± 2.4 × 10^−6^; 5.7 × 10^−7^ ± 4.2 × 10^−7^; respectively) and by *sst5TMD4* ≳ *SSTR5* ≳ *SSTR1* in U-118 MG (2.6 × 10^−6^ ± 1.7 × 10^−6^; 1.3 × 10^−6^ ± 7.1 × 10^−7^; 6.0 × 10^−7^ ± 1.4 × 10^−7^; respectively) (Figure 5A). Based on the present results indicating that *SSTR2* is the dominant receptor (followed by *sst5TMD4* and *SSTR5* in both cell models), together with previous results demonstrating that sst5TMD4 can physically interact with SSTR2 and SSTR5 and alter their signaling [15], we next interrogated whether the overexpression of *sst5TMD4* in GBM cells could be capable of modulating the expression levels of *SSTR2* and *SSTR5*. Interestingly, we found that the expression levels of *SSTR2*, but not *SSTR5*, were significantly decreased when *sst5TMD4* was overexpressed in both GBM cell models (Figure 5B).

Finally, we demonstrated that proliferation rate of GBM cells was not altered in response to the treatment of any of the available SSAs [i.e., first generation (octreotide or lanreotide) and second generation (pasireotide)] under normal-basal conditions (Figure 5C). In contrast, when *sst5TMD4* expression was silenced, pasireotide treatment was able to significantly decrease the proliferation rate in both GBM cell models at 24 h (Figure 5C), suggesting that a reduction in the levels of *sst5TMD4* could serve to sensitize GBM cells to pasireotide treatment.

## 3. Discussion

GBM is the most common CNS malignant tumor type, which represents a critical and relevant problem worldwide. GBM is associated with a high recurrence rate, a devastating prognosis (with a survival generally less than 5% in 5 years from diagnosis), and an elevated cost for the health systems [1,34,35]. Important progress in GBM medical management has been possible in recent years [34,36]; however, the existing strategies are still very limited, highlighting the critical need to identify novel avenues for GBM patients, to improve diagnose, to predict their tumor behavior and prognosis, but specially to provide useful tools to develop novel therapeutic approaches.

In this context, the development of SSAs as therapeutic opportunities has revolutionized the clinical management of patients with certain endocrine pathologies, and nowadays are considered the mainstay in the medical management of some pituitary and neuroendocrine tumors [24,25,37]. However, SSAs are frequently ineffective in a subset of patients, suggesting that key molecular determinants could be essential for the response to this pharmacological treatment [38]. In fact, SSTRs are expressed in brain tumors [1,2], but attempts to apply SSAs have rendered inconclusive results, since the limited studies available did not report a clear therapeutic value but the mechanistic reasons of those experimental failures are still unknown [26,27,28,30,39]. Given that aberrant alternative splicing is one of the hallmarks of cancer (including GBM) [8,40,41], and our group has demonstrated the overexpression and relevant pathological function of the splicing variant *sst5TMD4* in different tumor pathologies [14,15,16,17,18,19,20,21,22], we hypothesized that sst5TMD4 could be expressed and might play an oncogenic role in GBM pathophysiology and/or in the poor response to SSAs observed in GBM.

Our results demonstrated, for the first time, that sst5TMD4 is overexpressed (at mRNA and protein levels) in GBM samples compared with non-tumor brain samples and/or anaplastic (grade III) astrocytoma samples. Indeed, ROC-curve analysis revealed that *sst5TMD4* expression could clearly discriminate between GBM samples and control or anaplastic astrocytoma samples, suggesting that sst5TMD4 might be useful as a new diagnostic biomarker of GBM. Consistent with this idea, we found that lower *sst5TMD4* expression in GBM patients tended to be associated with *IDH1*mut (which is classically linked with better prognosis, survival, and a low-level necrosis in gliomas [32]) and non-recurrent tumors, but most importantly, we found that it was significantly associated with a better survival rate. Therefore, these results also suggest that sst5TMD4 might represent a new prognostic biomarker of progression and survival, and that it might also exert an oncogenic role in GBM biology. These observations compare favorably with previous reports indicating that the expression of this splicing variant is consistently increased in several endocrine-related tumors compared with control tissues, and sst5TMD4 expression associates with poor prognosis and/or aggressiveness features in these tumor pathologies, including pituitary [14,17], neuroendocrine [19], breast [15,20], prostate [22] and thyroid [16,18] tumors. Based on these observations, it is not unreasonable to suggest that sst5TMD4 overexpression might be a common cellular/molecular hallmark associated to an aberrant splicing process of the *SSTR5* receptor gene across various tumor pathologies. In line with this, we have recently demonstrated that the splicing machinery is drastically dysregulated in GBM which could represent a new source for the identification of novel diagnostic, prognostic, and therapeutic targets in GBM [8]. In fact, ours and other groups have previously demonstrated that GBM is characterized by the presence of several aberrant splicing variants (e.g., of *TP73*, *GLI1*, MAPKs, growth factor receptors, matricellular proteins, etc. [8,42,43]), reinforcing the idea that an aberrant alternative splicing could be also one of the hallmarks of GBM as happens in other cancer types.

Therefore, based on these data, we next explored the direct functional role of sst5TMD4 in GBM cells. We found that overexpression of *sst5TMD4* increased proliferation and/or migration in GBM cells, whereas, in contrast, *sst5TMD4* silencing decreased these two functional parameters. However, it should be mentioned that the proliferative actions seemed to be cell line dependent (i.e., proliferation was altered in U-87 MG but not in U-118 MG cells), which might be due to specific phenotypic differences between the two GBM cell lines used (i.e., mutation profile, aggressiveness, metabolic rate, etc. [33]). As discussed below, these dissimilarities could be also explained by the signaling pathways and mediators found in the present study to be differentially linked to *sst5TMD4* overexpression in both GBM cells (e.g., overall activation of NF-κB-pathway [44,45], as well as the specific activation of CDKN1B [44] in U-87 MG cells). In fact, this observation could also suggest a differential role of sst5TMD4 in different stages of aggressiveness, being its oncogenic role (at least proliferative role) possibly more pronounced in more aggressive and metabolically active cells (i.e., U-87 MG). In this sense, similar divergences in response to sst5TMD4-system modulation have been found in other tumor cell models [i.e., neuroendocrine (BON-1 vs. QGP-1 cells), breast (MDA-MB-231 vs. MCF-7 cells), and prostate (PC-3 vs. 22Rv1 cells) [19,21], which have been also attributed to the distinct nature of the cell models. Nonetheless, our data clearly demonstrate that *sst5TMD4* splicing variant is functionally active in GBM cells and that its presence is directly associated with their progression and aggressiveness features.

To investigate the putative molecular mechanisms involved in the oncogenic actions of sst5TMD4 in GBM, we used U-87 MG and U-118 MG cells overexpressing *sst5TMD4*. We found that sst5TMD4 could exert its function through the activation of multiple critical mediators of various cancer-relevant signaling pathways closely associated with malignancy progression in different tumor pathologies [8,44,45,46,47,48,49], such as an activation of AKT (i.e., AKT1, BAD, EIF4EBP1, P70S6K, PDK1, GSK3A, CDKN1B, and PRASA40), JAK/STAT (i.e., SRC, STAT1, STAT2, and STAT3), NF-κB (i.e., ATM, EIF2A, HDAC2, TAK1, TBK1, and ZAP70), and TGF-β (i.e., FOS, JUN, ATF2, FOS, JUN, SMAD1, SMAD4, and SMAD5) pathways. Moreover, we also found an overall inactivation of multiple mediators of the MAPK pathway (i.e., JNK, MEK, MMK3, MMK6, TP53, RPS6KA1, RPS6KA3, ERK1/2, MSK2, and MAPK14) in *sst5TMD4*-overexpressed GBM cells, which might be also associated with the oncogenic actions of this splicing variant in GBM cells since MAPK inactivation (including the well-known tumor suppressor TP53) has been linked to an inhibition of apoptosis in order to enhance cell survival [50,51,52,53].

Finally, the results of this study open a new research avenue in the study of GBM in that attempts to apply SSAs in GBM patients have rendered inconclusive results, wherein a poor response to SSAs has been shown to be associated with the presence of sst5TMD4 in some tumor pathologies [14,17,18,22,23]. In this sense, to the best of our knowledge, this is the first report demonstrating that under basal conditions U-87 MG and U-118 MG GBM cells, although expressing significant levels of *SSTR2* and *SSTR5* (the primary targets for the available SSAs), are not responsive, in terms of cell proliferation, to SSAs treatment [first generation (octreotide and lanreotide, which preferentially bind to SSTR2 and, to a lesser extent, to SSTR5) and second generation (pasireotide, that preferentially binds to SSTR5 and, to a lesser extent, to SSTR2) [52]]; however, the mechanisms of this SSA-resistance had not been yet explored in GBM cells. Interestingly, we found that overexpression of *sst5TMD4* significantly reduced *SSTR2* expression in both U-87 MG and U-118 MG cells, which might explain why GBM cells respond poorly to SSAs. Indeed, we have previously demonstrated that sst5TMD4 can physically interact with SSTR2 and disrupt the function of other SSTRs (mainly SSTR2 and SSTR5) by inhibiting the ability of the cells to respond to SSAs [12,14,15,17,22]. Therefore, based on all these data, it is tempting to speculate that a potential functional association between SSTR2, SSTR5 and sst5TMD4 could also exist in GBM, and that the high *sst5TMD4* expression levels found in tissues/cells from GBM patients could help, in part, to explain the inefficacy of SSA therapy in the scarce and limited GBM trials implemented hitherto [29,39,54,55,56]. Obviously, further work will be required to evaluate if the presence of sst5TMD4 might interfere with the effect of SSAs in GBM cells but, in support of this idea is the fact that we found in the present study that *sst5TMD4*-silencing was able to significantly sensitize GBM cells to the antiproliferative effect of pasireotide in both U-87 MG and U-118 MG cells.

## 4. Materials and Methods

### 4.1. Reagents

Unless otherwise indicated, reagents and products were purchased from Sigma-Aldrich (St. Louis, MO, USA). siRNA and plasmid of *sst5TMD4* were precisely designed as previously reported [12,22].

### 4.2. Patients and Samples

A total of 56 tumor samples were obtained by intracranial surgery from patients previously diagnosed with anaplastic astrocytoma (*n* = 9) or GBM (*n* = 47) from the Reina Sofia University Hospital (Table 1). This study was conducted in accordance with the ethical standards of the Helsinki Declaration, of the World Medical Association and with the approval of the Hospital Ethic Committee. Written informed consent was obtained from all individuals included in the study. Control (non-tumor) brain tissue samples (*n* = 20; Table 1) were obtained from four healthy donors [autopsy; four different brain areas (Brocca, Wernicke, cingulate, and medial) of 4 patients; *n* = 16], and from four epilepsy patients (from lobectomy surgery), as previously described [8]. All samples were histologically studied by an expert pathologist to confirm the GBM, anaplastic astrocytoma, and the non-tumor samples. Demographic and clinical characteristics of all patients/donors were collected to carry out clinical correlations (Table 1).

### 4.3. Immunohistochemical Analysis

sst5TMD4 IHC analysis was performed using a custom-made and previously validated polyclonal rabbit anti-human sst5TMD4 antibody (clone name: 66498; 1:100) [12,22], which was specifically designed to target the differential region between sst5TMD4 and SSTR5 proteins (CRERLSGHKSWQEKG). In particular, sst5TMD4 protein expression was analyzed on FFPE tissue samples from controls (non-tumor brain samples, *n* = 4) and from GBM (*n* = 5). Briefly, as previously described [12,15,17,22], samples were incubated with the specific antibody in deparaffinized sections overnight at 4 °C, followed by incubation with the appropriate horseradish peroxidase–conjugated secondary antibody (Envision system; Dako, Barcelona, Spain). Finally, sections were developed with 3,39-diaminobenzidine (Envision system 2-Kit Solution DAB), contrasted with hematoxylin, and mounted in an automatic mounter (Tissue-Tek Film; Sakura, Japan). Finally, a specialist pathologist performed a histopathologic analysis of the tumors following a blinded protocol. In the analysis, +, ++, +++ mean low, moderate, and high intensities of tumor region staining compared with the normal-adjacent region, respectively.

### 4.4. Cell Cultures

GBM cell lines (U-87 MG and U-118 MG) were obtained from the American Type Culture Collection (ATCC: #HTB-14/#HTB-15, respectively) and cultured (passages < 20) according to the supplier’s recommendations. These cell lines were previously checked for mycoplasma contamination by PCR every month, as previously reported [57].

### 4.5. Modulation of the Levels of sst5TMD4 Expression (Overexpression and Silencing) in GBM Cells

As previously reported [15,22], U-87 MG and U-118 MG cells (200,000 cells/well) were stably transfected with pCDNA3.1 vector (ThermoFisher Scientific, Waltham, MA, USA) that contained the empty vector (control) or the *sst5TMD4* transcript using Lipofectamine 3000 (ThermoFisher Scientific, Waltham, MA, USA), according to the manufacturer’s instructions. After 24 h, medium was refreshed and next day, selection antibiotic geneticin at 0.01% was added with a confluence of 80–90%. Then, 24–48 h later, non-stable transfected cells started to die due to the geneticin action and *sst5TMD4*-stable cells were selected and maintained for an additional 5–6 weeks, refreshing medium with geneticin. Stable transfection of *sst5TMD4* was confirmed (Supplemental Figure S2A) by quantitative-PCR (qPCR). Then, cells were seeded for different functional assays in response to *sst5TMD4* overexpression [proliferation and migration at different time points (e.g., 6, 24, 48 and 72 h after an initial start point (0 h, moment when the same numbers of starved mock and *sst5TMD4*-stable transfected cells were seeded in each well); see below].

Moreover, a pre-designed and previously validated specific small interfering RNA oligo (siRNA; 5’-CACAAAUCCUGGCAGGAGATT-3´; [22]) for knockdown of endogenous levels of *sst5TMD4* was used, along with the SilencerVR Select Negative Control siRNA (#4390843, ThermoFisher Scientific, Waltham, MA, USA) as a scramble control. Specifically, U-87 MG and U-118 cells (150,000 cells/well) were transfected with 10^−7^ M of each siRNA individually using Lipofectamine RNAiMaxVR (#13778-075, ThermoFisher Scientific, Waltham, MA, USA) according to the manufacturer’s instructions. After 24 h, cells were collected for validation of the specific silencing of *sst5TMD4* [by qPCR; Supplemental Figure S2B; without altering *SSTR5* expression (Supplemental Figure S2C)], and seeded for different functional assays (proliferation and migration; see below) in response to *sst5TMD4* silencing.

### 4.6. Measurements of Cell Proliferation

As previously described [22], cell proliferation in response to the modulation of *sst5TMD4* expression (overexpression and silencing) was estimated using Resazurin reagent (#R7017, ThermoFisher Scientific, Waltham, MA, USA), after seeding 5000 cells per well (both U-87 MG and U-118 MG cells) in a 96-well plate. Additionally, proliferation rate was also studied (U-87 MG and U-118 MG cells) in response to the treatment with different SSAs (octreotide, lanreotide, and pasireotide, 10^−7^ M) under basal conditions or in response to *sst5TMD4* silencing. Briefly, cells were seeded in 96-well culture plates and serum-starved for 12–16 h in order to achieve cell cycle synchronization. Then, cell proliferation was measured at 0, 24, 48, and 72 h using Flex Station III (Molecular Devices, Madrid, Spain).

### 4.7. Measurement of Cell Migration Capacity

For the migration assay, 150,000 cells/well (U-118 MG) were cultured under confluence and serum-starved for 24 h to achieve cell synchronization, and then, the wound was made using a 200 µL sterile pipette tip. Wells were rinsed and cells incubated for 6 and 24 h with supplemented medium without FBS. Wound healing was normalized with the area just after the wound was performed. Three pictures were randomly acquired along the wound per well to calculate the area by ImageJ software v.1.49 [58].

### 4.8. RNA Extraction, Retrotranscription, and Gene Expression Measurement by qPCR

Total RNA from human samples was extracted followed by DNase treatment using the AllPrep DNA/RNA/Protein Mini Kit and the RNase-free DNase set (#80004/#79254, Qiagen, Hilden, Germany), respectively. Total RNA from GBM cell lines was extracted with TRIzolVR Reagent (#15596026, ThermoFisher Scientific, Waltham, MA, USA). In both cases, total RNA concentration and purity were assessed by Nanodrop One Microvolume UV-Vis Spectrophotometer (ThermoFisher Scientific, Waltham, MA, USA). Total RNA was retrotranscribed by using random hexamer primers and the RevertAid RT Reverse Transcription Kit (#K1691, ThermoFisher Scientific, Waltham, MA, USA). Thermal profile and qPCR analysis to obtain absolute mRNA copy number/50 ng of sample of selected genes (*sst5TMD4*, *SSTR1*, *SSTR2*, *SSTR3*, *SSTR4*, and *SSTR5*) are reported elsewhere [12,18]. Human primers of the target genes used in the study were specifically designed with the Primer3 software v.0.4.0 (Supplementary Table S1). To control for variations in the efficiency of the retrotranscription reaction, mRNA copy numbers of the different transcripts analyzed were adjusted by the expression level of a housekeeping gene [b-actin (*ACTB*; in the case of human samples) or *GAPDH* (in the case of cell lines)], where mRNA levels of these housekeeping genes did not significantly vary between experimental groups (data not shown).

### 4.9. Human Phosphorylation Pathway Profiling Array

The phosphorylation pathway profiling array was performed using the Human Phosphorylation Pathway Profiling Array C55 kit (Raybiotech, Inc. #AAH-PPP-1-4) to analyze the differential phosphorylation levels of 55 proteins belonging to 5 key oncogenic pathways (MAPK, AKT, JAK/STAT, NF-κB, and TGF-β) in response to *sst5TMD4* overexpression, following the manufacturer’s instructions and protocols. Cells were collected to obtain the whole protein with a specific lysis buffer with phosphatase inhibitors from a phosphoarray kit (#AAH-PPP-1-4, Raybiotech, Inc., Norcross, Georgia). Transient transfection was confirmed by qPCR (Supplemental Figure S2D). Briefly, membranes were incubated with blocking buffer at 25 °C for 30 min and then incubated overnight at 4 °C with 1 mL of U-87 MG and U-118 MG cell lysates [2.5 fold dilution; 100,000 cells/well; *n* = 3 of mock-transfected controls (pCDNA3.1+ plasmid) and *sst5TMD4*-overexpressing cells]. Then, the membranes were incubated with Detection Antibody Cocktail at room temperature for 2 h and, next with horseradish peroxidase (HRP)-labeled anti-rabbit secondary antibody at room temperature for 2 h. The signals were collected after adding ECL reagent by a chemiluminescence detection system (GE Healthcare). A densitometric analysis of the array spots was carried out with ImageJ software v.1.49 using positive control spots as a normalizing factor.

### 4.10. Bioinformatic and Statistical Analysis

Data were evaluated for heterogeneity of variance by using the Kolmogorov–Smirnov test. Statistical differences were assessed by Mann–Whitney U-test, T-test, or One-Way ANOVA. In survival analysis, groups were selected based on the cut-off points determined by survminer R package (R language v.4.1), and Log-Rank and Gehan–Wilcoxon tests were performed. Correlations were studied using the Pearson and Spearman correlation tests. All statistical analyses were performed using Prism software v.8.0 (GraphPad Software, San Diego, CA, USA). Values of *p* < 0.05 were considered statistically significant. A trend was considered when *p* ≤ 0.10. Data represent means ± SEM. (+) *p* ≤ 0.10, (*) *p* < 0.05, (**) *p* < 0.01, (***) *p* < 0.001, compared to control conditions (scramble/mock). Log2 (Fold Change) of ±0.2 was considered as a threshold in Human Phosphorylation Pathway Profiling Array. Circle plots were implemented in R language v.4.1 using the following packages: tidyverse, Viridis and ggplot2.

## 5. Conclusions

Taken together, our results unveiled new conceptual and functional avenues in GBM with potential clinical implications, by demonstrating that sst5TMD4 is overexpressed in GBM and associated with GBM survival/progression and key pathophysiological processes in GBM biology (i.e., proliferation and migration capacity), likely by modulating different oncogenic signaling pathways (AKT/JAK-STAT/NF-κB/TGF-β). Moreover, our study demonstrated that the modulation of sst5TMD4 expression levels could be putative therapeutic avenue that should be explored in the future in GBM since its silencing decreased proliferation and migration rates in GBM cells and sensitized these cells to the antitumor effect of pasireotide; however, further investigations will be required to firmly test the value of sst5TMD4 as a therapeutic target. Therefore, these results point out sst5TMD4 as a useful diagnostic and prognostic biomarker, and as a potential target in the future development of therapeutic approaches in GBM patients, offering a clinically relevant opportunity that should be tested for use in humans.

## Figures and Tables

**Table 1:** Demographic and clinical parameters of patients with glioblastoma (GBM; *n* = 47) and anaplastic astrocytoma (*n* = 9) included in this study. Non-pathologic brain control samples from donors (*n* = 8) were obtained from autopsies [4 patients; 4 different brain areas (Brocca, Wernicke, cingulate, and medial)/patient; total of 16 samples] or epileptic patients (from lobectomy surgery; *n* = 4).

Parameters	Control Patients	Anaplastic Astrocytoma	Glioblastoma
Patients (*n*)	8	9	47
Gender (M/F)	2(25%)/6(75%)	3(33.3%)/6(66.7%)	19(40.4%)/28(59.6%)
Age at surgical intervention (mean ± desvest)	44.13 ± 4.32	51.9 ± 13.0	56.9 ± 13.2
% Ki67 (mean ± desvest)	-	15.2 ± 3.2%	28 ± 14.6%
% of *TP53* positive	-	87.5%	81.8%
% of *IDH1* positive	-	33%	11.6%
% of recurrent tumors	-	0%	18%

## Data Availability

The datasets generated and/or analyzed during the current study are available from the corresponding author upon reasonable request.

## Supplementary Materials

The following are available online at https://www.mdpi.com/article/10.3390/ijms23031143/s1.
