# Supplementary material for: Somatostatin Receptor Splicing Variant sst5TMD4 Overexpression in Glioblastoma Is Associated with Poor Survival, Increased Aggressiveness Features, and Somatostatin Analogs Resistance
Source: Int J Mol Sci. 2022 Jan 20;23(3):1143. doi: 10.3390/ijms23031143 (PMC8835306; doi:10.3390/ijms23031143)
Supplement: Supplementary file 1 [file ijms-23-01143-s001.zip › ijms-1521347-supplementary.pdf]

Supplemental Figure S1

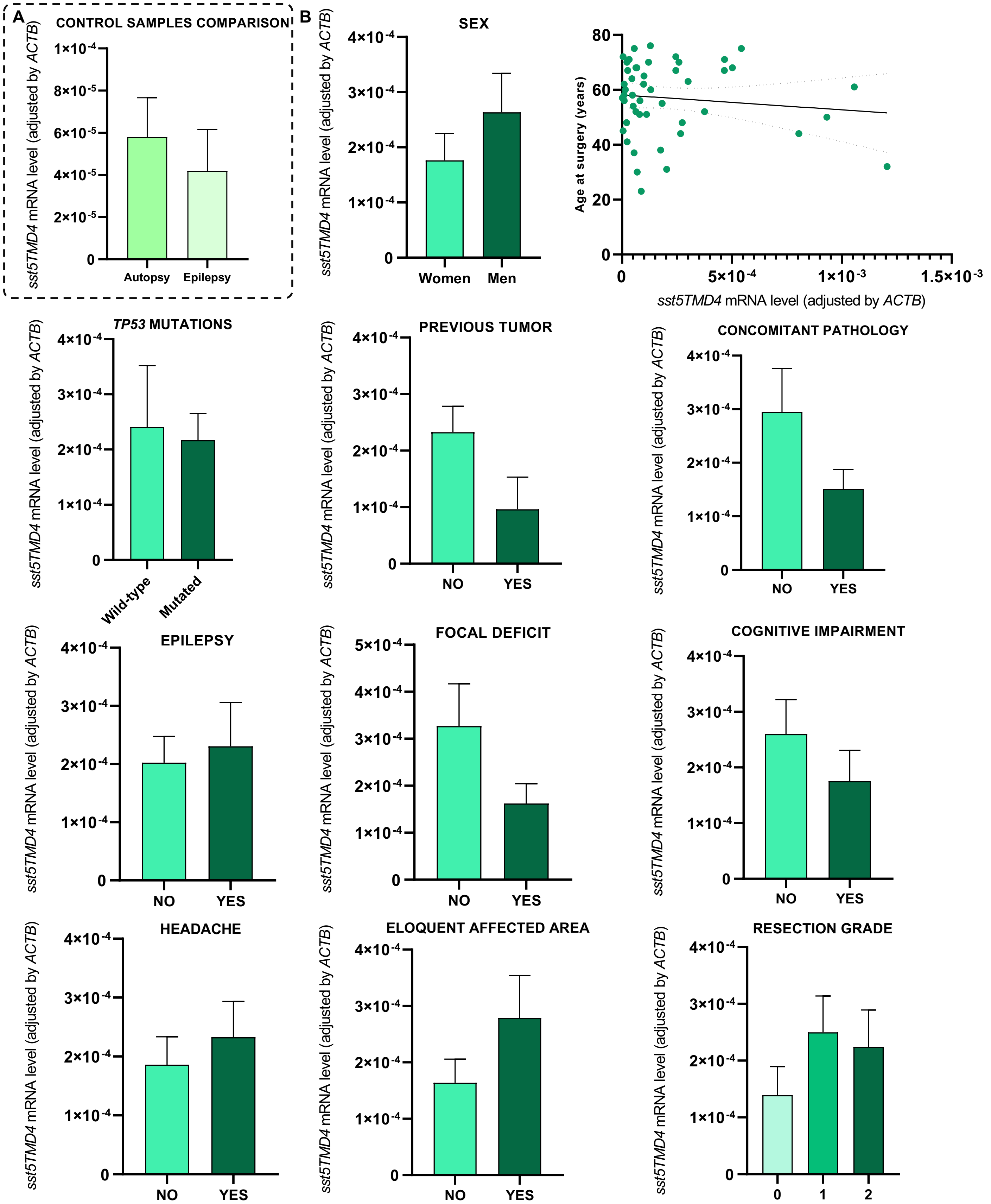

Supplemental Figure S1. Comparison of *sst5TMD4* mRNA expression between two different control sample groups (first figure, left top corner) and relation between *sst5TMD4* mRNA levels and different clinical factors in glioblastoma patients (other figures). Non-tumor control brains were obtained from autopsies or epileptic patients (lobectomy). Clinical parameters measured in GBM samples were *TP53* mutations, age, sex, previous tumors, concomitant pathologies, epilepsy, focal deficit, cognitive impairment, headache, eloquent affected area, and resection grade.

Supplemental Figure S2

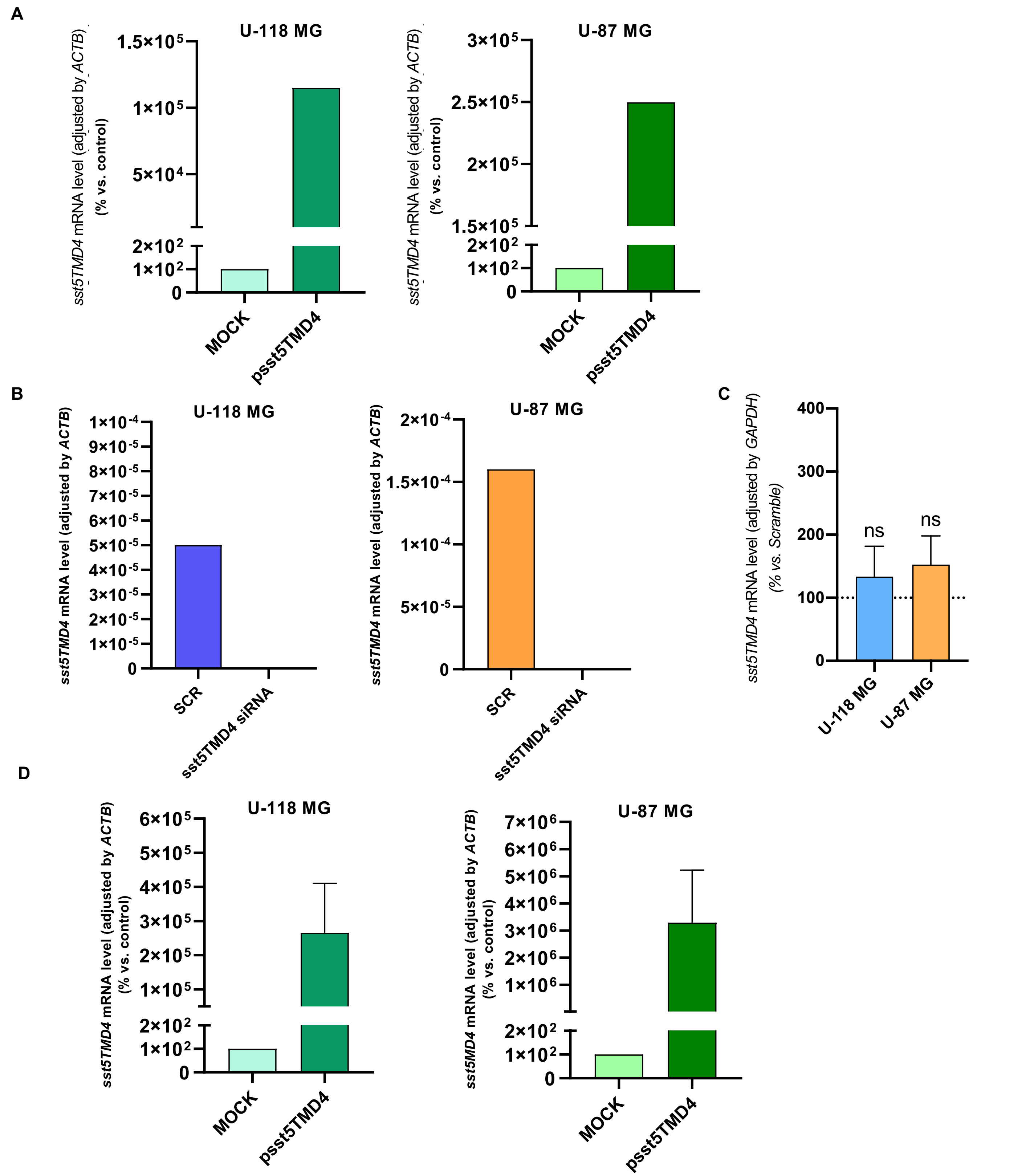

Supplemental Figure S2. Validation of the modulation of *sst5TMD4* expression in U-87 MG and U-118 MG cell lines. (A) Validation of stable overexpression of *sst5TMD4* in cells used for the functional assays. (**B)** Validation of transient silencing of *sst5TMD4* in cells used for the functional assays and (**C)** validation of the possible off-target *SSTR5* mRNA expression. (**D)** Validation of transient overexpression of *sst5TMD4* in cells used for the functional assays for the signaling assay (phosphorylation array).

Supplemental Table S1

Supplemental Table S1. Specific primers for human transcripts used in this study [including the two housekeeping genes (HK)] were specifically designed for quantitative real-time PCR. The official name of the genes, NCBI accession number of the transcripts, primers sequences, and product sizes of the amplification products are included.

	Gene	Genbank Accession number	Sense sequence	Antisense sequence	Amplicon size (pb)
HKs	***ACTB***	NM001101	ACTCTTCCAGCCTTCCTTCCT	CAGTGATCTCCTTCTGCATCCT	176
	GAPDH	NM_001256799	AATCCCATCACCATCTTCCA	AAATGAGCCCCAGCCTTC	122
SSTR FAMILY	***SSTR1***	NM_001049	CACATTTCTCATGGGCTTCCT	ACAAACACCATCACCACCATC	165
	SSTR2	NM_001050	GGCATGTTTGACTTTGTGGTG	GTCTCATTCAGCCGGGATTT	185
	SSTR3	NM_001051	TGCCTTCTTTGGGCTCTACTT	ATCCTCCTCCTCAGTCTTCTCC	190
	SSTR4	NM_001052	TCTTTGTGCTCTGCTGGATG	GGATAAGGGACACGTGGTTG	174
	SSTR5	NM_001053	CTGGTGTTTGCGGGATGTT	GAAGCTCTGGCGGAAGTTGT	183
	sst5TMD4	DQ448304 (only uniprot annotation)	TACCTGCAACCGTCTGCC	AGCCTGGGCCTTTCTCCT	98
